# A data-driven methodology to discover similarities between cocaine samples

**DOI:** 10.1038/s41598-020-72652-w

**Published:** 2020-09-29

**Authors:** Fidelia Cascini, Nadia De Giovanni, Ilaria Inserra, Federico Santaroni, Luigi Laura

**Affiliations:** 1 Department of Life Sciences and Public Health, Università Cattolica del Sacro Cuore, 00168 Rome, Italy; 2 Fondazione Policlinico Agostino Gemelli IRCCS, Largo Agostino Gemelli 8, 00168 Rome, Italy; 3 Department of Computer, Control, and Management Engineering Antonio Ruberti (DIAG), Sapienza University of Rome, 00186 Rome, Italy; 4 International Telematic University Uninettuno of Rome, Rome, Italy

**Keywords:** Computational models, Data mining, Data processing, Databases, Machine learning

## Abstract

Machine learning has been used for distinct purposes in the science field but no applications on illegal drug have been done before. This study proposes a new web-based system for cocaine classification, profiling relations and comparison, that is capable of producing meaningful output based on a large amount of chemical profiling’s data. In particular, the Profiling Relations In Drug trafficking in Europe (PRIDE) system, offers several advantages to intelligence actions across Europe. Thus, it provides a standardized, broad methodology which uses machine learning algorithms to classify and compare drug profiles, highlight how similar drug samples are, and how probable it is that they share a common origin, batch, or preparation process. We evaluated the proposed algorithms using precision and recall metrics and analyzed the quality of predictions performed by the algorithms, with respect to our gold standard. In our experiments, we reached a value of 88% for F_0.5_-measure, 91% for precision, and 78% for recall, confirming our main hypothesis: machine learning can learn and be applied to have an automatic classification of cocaine profiles.

## Introduction

Machine learning has shown an increased use in medicine and healthcare during recent years. It’s use has been noted with machine learning tools demonstrating their significance, especially for automatic classification, quantification, detection, or even diagnosis processes^1^. Examples of machine learning’s benefits have been explored across different research studies, showing its utility for automated examination and quantification of histopathological slides^2^, automatic analysis of IgA-class EmA test for celiac disease^3^, as well as others^4^.

Future applications in the field show additional possibilities for machine learning applications. The discovery of a drug’s similarities is an innovative and promising approach to control illegal drug trafficking and, consequently, prevent drug consumption. Social purposes, public health and investigative intelligence can benefit from effective actions against drug supply and diffusion for public health, such as a quick chemical profiling and real time traceability of samples. These comparisons can then be applied to establish distribution and trafficking links between multiple seizures and samples found in different locations or which have been in the possession of different individuals.

Different parameters can be detected by analysing cocaine samples, thus obtaining comparable profiles of the substance. A process commonly defined as “chemical profiling” typically, and which involves several analyses designed to produce a detailed picture of the drug sample (usually in the form of chromatographic data). Cocaine can also be classified through chemical profiles—also known as ‘signatures’ or ‘impurity profiles’—resulting in the identification and the quantification of major and minor components such as alkaloids, solvents, diluents and adulterants^5^.

During cocaine production, *Erythroxylum* plants are used, passing through a laborious process that can add impurities to the final product. The easiest way to detect impurities usually belongs to one of the following categories: (1) Compounds co-extracted from the raw plant materials; (2) By-products resulting from transformation reactions of alkaloids during the process; (3) Solvents and reagents arising from laboratory processing; (4) Adulterants and diluents added at any point in the distribution chain.

During the production and purification of cocaine, some by-products can result from the hydrolysis, oxidation or thermo-degradation processes^6^. Moreover, the solvents employed during the cocaine production (including the conversion of a cocaine base to cocaine hydrochloride) provide a lot of information about the processing. In fact, the entire process consists of three main steps, starting with a first step of alkaloid extraction from the harvested plants to produce the coca ‘pasta’. This step is followed by the coca ‘pasta’s transformation to cocaine in its freebase form. The last step is the crystallization of cocaine in a salt form (hydrochloride: HCl), which gives a crystal matrix containing occluded solvents.

In this paper, we present a data-driven approach to discover similarities among profiles of cocaine samples. We investigated drug samples from cocaine seizures of more than 5 kg and investigated chemical markers such as residual solvents and by-product impurities to profile and compare substances.

The added value of this research is the coupling of two different aspects. The first is the accurate characterization of cocaine seizures by the analytical detection of cocaine purity, quali-quantitative identification of origin impurities and by-products, as well as determination of occluded solvents. These parameters, obtained with highly specific and sensitive techniques using validated methodologies, offer reproducible information so contributing to a reliable fingerprint of each analyzed sample. The second is the collection of all of the analytical data (profiles) from cocaine samples and the study of the correlations between them with high performances that can be achieved by the means of a specific algorithms. In particular, a platform continuously updated with cocaine profiles was designed to collect information over time. This platform can efficiently support people working against the cocaine drug market because it acts like a smart analyst. It can collect and process large amounts of information (from analytical to circumstantial data) and see the relationships between them. It tells us how similar drug samples are, and how probable it is that they share a common origin, batch or preparation process. It can receive data from an unlimited range of connection sources in real time and then easily share the output at both a national and international level.

## Results

In this section, we describe our main results in terms of the process we developed, analysis methodology, algorithms, and related performances.

We present a process which, given the chemical analysis of cocaine samples, uses a machine-learning algorithm to automatically detect samples in the database which come from the same production process. This process has been designed assuming the database has samples collected from all of the European countries. Indeed, this process might support law enforcement by providing a better understanding of the origin of cocaine samples and allow agencies to coordinate efforts in case the system detects similarities in two seizures performed by two different law enforcement departments. Note that a manual approach- i.e., domain-expert driven- may be useful only in the case of analysis and comparison performed on a small set of samples. An automated algorithm can instead find correlations which might be very hard, or even impossible, to manually find in a large set of samples. We have experimentally tested our algorithms against a set of real-world data, verifying that it is capable of providing reliable predictions. For our experimental purposes, we relied on a set of 148 samples coming from 40 different real seizures performed by law enforcements in Italy from 2014 to 2018.

### The process

**Figure 1:**
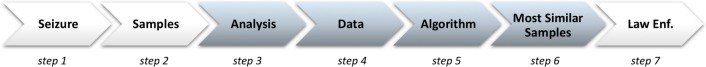
The process. The process of using machine-learning to automatically detect cocaine samples. A seizure (Step 1) usually provides different samples (Step 2) extracted from different packs of a cocaine seizure. Samples are analyzed by laboratories (Step 3) in order to produce data useful to predict similarities among samples (Step 4). Our algorithm (Step 5) processes data to try and find a sample in the database which seems to have been produced by the same process (Step 6). We then report relevant data and metadata to law enforcement to support the investigation and cooperation phase (Step 7).

In the process that we designed (Fig. 1), when a seizure occurs (Step 1), we typically find different batches of cocaine, possibly containing cocaine of different quality from different sources. From each pack in a batch, a sample is extracted (Step 2). All samples are then sent to laboratories for a chemical analysis (Step 3). The output of the chemical analyses provides the data (Step 4) required to detect similarities against samples already present in our database. We collect data in a database together with related metadata describing the seizure. Our algorithm (Step 5) processes the data and, for each sample, finds the best match (if existent) for which we can confidently state comes from a very similar production process. If a match is found (Step 6), we provide law enforcement the relevant data and metadata useful to the investigation phase. For example, the relevant data could include whether there is a different law enforcement department which is already investigating a seizure containing samples coming from the same production process (Step 7). We remark that at the end of the process, the new samples will be part of the database in order to increase the knowledge base for future discoveries.

### Performing chemical analysis

The process is based on chemical analysis because it can produce a reliable fingerprint of the chemical structure of samples. During the process, selected substances which come from the extraction and purification process of the drug, or from the change of the original alkaloids of the plant, are used as parameters for computing real samples. These have been identified in the laboratory using highly specific and sensitive techniques, thus offering reproducible information.

The laboratory process aims at measuring the impurities of cocaine and reporting the concentration of specific compounds in the sample, categorized into three groups: alkaloids and by-products; solvents and reagents; adulterants and diluents. For our purposes, we focus on solvents, alkaloids and by-products involved in the production process. This is because they constitute a large enough set of information to identify a specific cocaine production process, which may be also the signature of a specific producer. Additionally, experiments in this research have exposed that these data are able to be used to cluster samples into groups with closely related production processes.

As stated previously, data considered from the chemical analysis are those provided by solvents, alkaloids and by-products listed in (Table 1).

**Table 1:** Compounds considered in the approach.

Compounds	Unit	Compounds	Unit
Ethanol	ng/mg	Propyl_acetate	ng/mg
Acetone	ng/mg	Heptane	ng/mg
Chloro_methane	ng/mg	Methyl_cyclohexane	ng/mg
2-propanol	ng/mg	Ethyl_cyclohexane	ng/mg
Diethyleter	ng/mg	Toluene	ng/mg
Methyl_acetate	ng/mg	m-xylene	ng/mg
npropanol	ng/mg	o-xylene	ng/mg
Pentane	ng/mg	p-xylene	ng/mg
4-methyl-2-pentanone	ng/mg	1,2,4-trimethyl benzene	ng/mg
Dichloromethane	ng/mg	4-Methyl_benzaldehyde	µg/mg
2-methyl pentane	ng/mg	Methyl_benzoate	µg/mg
3-methyl pentane	ng/mg	4-Methyl_benzamide	µg/mg
Ethyl_acetate	ng/mg	Ecgonidine_methylester	µg/mg
Hexane	ng/mg	Ecgonine_methylester	µg/mg
Methyl_cyclopentane	ng/mg	Norcocaine	µg/mg
Benzene	ng/mg	Cocaethylene	µg/mg
2-methyl hexane	ng/mg	n-formyl_norcocaine	µg/mg
3-methyl hexane	ng/mg		

Data from the chemical analysis considered from the study are those provided by different solvents, alkaloids and by-products. 35 items are presented in the table.

### Computing most similar samples

**Figure 2:**
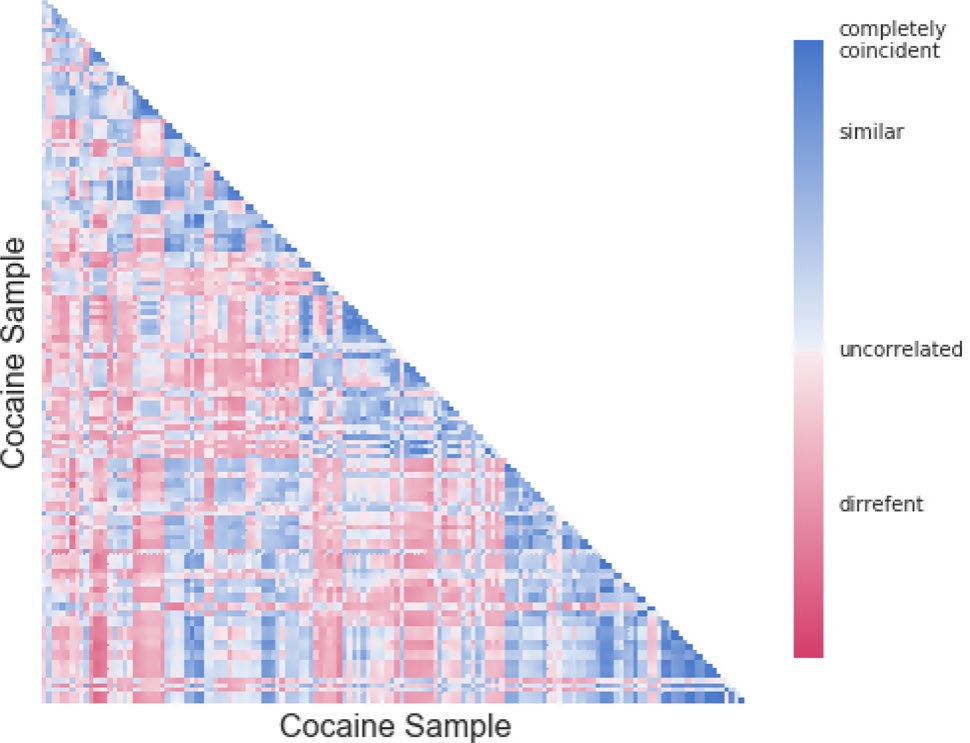
Similarity matrix of samples considered in our experiments. The matrix shows the similarity of samples considered in our experiments. In this graphical representation of the similarity matrix, each row and each column represents a cocaine sample, while the color of a cell (i, j) represents the similarity of the sample i with the sample j, according to the color code depicted on the right. Since similarity is symmetric, we only depict the lower triangular part of the matrix; we also avoid depicting the main diagonal, since a sample is always completely coincident to itself. In order to compute the most similar sample j to a given sample i, our algorithm considers only those candidates (blue cells) such that their similarity, s, (i, j) is sufficiently high to make us confident to predict that i and j come from the same production process. It follows that the most similar sample j to i, may not exist.

Given a sample *i,* our algorithm explores the concentrations of compounds listed in Table 1, in order to compute the most similar sample to *i* in the database. The algorithm makes use of a similarity function whose similarity matrix, applied to the set of samples used in our experiments, is depicted in Fig. 2. In this matrix, each row and each column represent a sample. The colour of cell (*i*,* j*) represents the similarity of samples *i* and *j,* according to the provided colour code, where blue denotes the samples which show interesting similarities.

During the first set of experiments, the algorithm considers all the samples in the database, with no concerns on the related seizures. Thus, it may happen that the most similar sample *j*, found for a given sample *i*, is in the same seizure of *i*. We call this algorithm “seizure-agnostic”. In Table 2, we report the main results from our experiments with the seizure-agnostic algorithm. As you can see, we can find the most similar sample of *122* samples out of the *148* provided (*82%*). The algorithm shows a precision of 93%, and a recall of 89% (see the discussion for more details). Nevertheless, in 61 cases, the given sample and its most similar sample turned out to belong to different seizures. This further highlights the effectiveness of the algorithm, since it can provide non-trivial correlations, in a seizure-agnostic scenario. Please see Table 2 below.

**Table 2:** Experimental results, seizure-agnostic algorithm.

Seizures	40
Samples	148
Most similar samples found	122
Precision	93%
Recall	89%
Most similar samples in a different seizure	61

Results of the experiments performed on the given set of samples (148 samples, coming from 40 seizures). In these experiments, the algorithm we used does not consider the seizure a sample belongs to. Thus, the most similar sample *j* found for a given sample *i* might be in the same seizure as *i*. Nevertheless, for 61 cases the given sample and its most similar sample belong to different seizures.

If there is special interest in finding correlations occurring between different seizures, the algorithm can be slightly modified to discard similarities found between samples of the same seizure. This algorithm has been named “Seizure-Aware”. In Table 3, the main results of the seizure-aware algorithm are reported.

**Table 3:** Experimental Results, Seizure-Aware Algorithm.

Seizures	40
Samples	148
Most similar samples found in different seizures	102
Precision	91%
Recall	78%

Results of the experiments performed on the given set of samples (148 samples, coming from 40 seizures). In these experiments, the algorithm does considers the seizure a sample belongs to, i.e., it discards similarities found among samples in the same seizure. In this setting in 102 cases we can find the most similar sample.

Note that, by discarding the similarities in the same seizures, we may no longer be able to find the most similar sample, while in some cases, we are still able to find a good match in a different seizure. Indeed, with the seizure-aware algorithm we can find the most similar sample coming from a different seizure in *102* cases (69%), with a negligible loss of precision (91%, compared to 93% in the seizure-agnostic algorithm), scarifying, as one can expect, a little recall (78%, compared to 89% in the seizure-agnostic algorithm).

## Discussion

The performance reliability of our algorithm has been verified experimentally. The algorithm allows its user to find useful and reliable correlations between different cocaine samples. Once the analysis methodology is established, it is capable of providing a reliable fingerprint of cocaine production by looking for similarities in the process. This characteristic is completely different from the manual approach. In fact, in the manual approach, given a pair of samples, a chemist-toxicologist (the domain expert) analyses the by-products, solvents and alkaloids data to understand whether they potentially come from the same production process. As a deficiency, there is no specifically recognized protocol for this task in the manual approach (details on this approach are available in the Supplementary Information section).

Although this approach is not rigorous, it may provide useful insights on the similarity between two samples. However, it can only state whether two samples are similar, dissimilar or potentially similar, it cannot provide a measurable degree of similarity. Thus, it cannot find the most similar sample. Moreover, although an expert may provide such insights on a given pair of samples, they can hardly analyze all possible pairs in a large collection of samples manually. From these considerations, it can be concluded that a manual approach cannot drive us towards our goal: computing the most similar sample to a given one, i.e., the sample in our database finds the best match (if it exists) in terms of production process.

To this end, we designed a reliable algorithm to compute the most similar sample to a given one. As commonly known, in order to evaluate the performances of an algorithm, in terms of its reliability in a machine-learning setting, we should have a so-called gold standard. In our scenario, a gold standard would be a collection of samples such that for each given pair in the collection, we know for sure whether they come from the same production process or not. However, we cannot use such a set of information. As a matter of fact, we do not have access to these data due to law enforcement restrictions. Additionally, for some of the seizures, law enforcement may have closed the investigation or not pursued investigations to the extent of rebuilding the entire production history of all seizures performed. On one hand, such a history would be a useful tool for the investigation phase. On the other hand, this is not always the final goal of law enforcement. Thus, we tried to overcome to this limit by building a synthetic gold standard by consulting domain experts. In particular, when our algorithm finds the most similar sample *j* to a given sample *i*, we follow the domain-expert approach previously described to understand whether the two samples can actually be considered similar or not. If the experts say the samples are potentially similar, we consider the prediction *j* to be correct, and we call this case *True-Positive* (*TP*). If the prediction *j* turns out to be incorrect, we say we have a *False-Positive* (*FP*). When the algorithm cannot find the best match, we define *True-Negative* (*TN*) and *False-Negative* (*FN*) analogously. In order to discuss the performances of our algorithm, let us introduce the metrics that we considered. We named *Recall* the ratio between True-Positives and the sum of True-Positives and False-Negatives, i.e., *TP/(TP* + *FN)*. Thus, in our scenario, the recall tells us how many correct best matches (*TP*) we found over all of the best matches we should have found (*TP* + *FN*). Similarly, we call *Precision* the ratio between True-Positives and the sum of the True-Positives and False-Positives, i.e., TP/(TP + FP). Thus, in our scenario, precision tells us how many correct predictions (TP) we made over all our predictions (TP + FP).

Our algorithm exploits a formally defined similarity function (see also the following paragraph on methods). This function computes the degree of similarities between samples considering the overall distribution of compound concentrations. However, the mere similarity function is not sufficient. We also require a similarity threshold, *th*, over which we can be confident to state that two samples may have been produced by the same production process. In other words, given a sample *i*, to look for the best match we first discard those samples having similarity to *i* less than *th*. An effective value for *th* must be learned from the data, choosing the value which leads the algorithm to the best performances. To this end, we remark that we are mainly interested in trusting our predictions (high precision), but we also want our algorithm to generalize well and lead us to non-trivial predictions, thus a reasonable recall is also necessary. Therefore, we determine our threshold value as the one which leads to the maximum value of the F_0.5_-measure of the algorithm. As a matter of fact, the F_0.5_-measure combines precision and recall through harmonic mean, putting more emphasis on precision than on recall. As a formula, F_0.5_-measure is defined as

$$F_{0.5} {\text{-}} measure = \left( {1 + 0.5^{2} } \right)\frac{{precision * recall}}{{0.5^{2} * precision + recall}}$$

We performed two sets of experiments using the seizure-agnostic and seizure-aware algorithms, where we looked for the best value for the threshold, according to our data and the resulting F_0.5_-measure, using a so-called grid-search methodology. In other words, we start from a threshold value of 0.1, and we increment it by 0.1 until we reach the final value 1.0. For each such value, we compute the resulting recall, precision, and F_0.5_-measure. Eventually, we choose the threshold value which maximizes the F_0.5_-measure.

**Figure 3:**
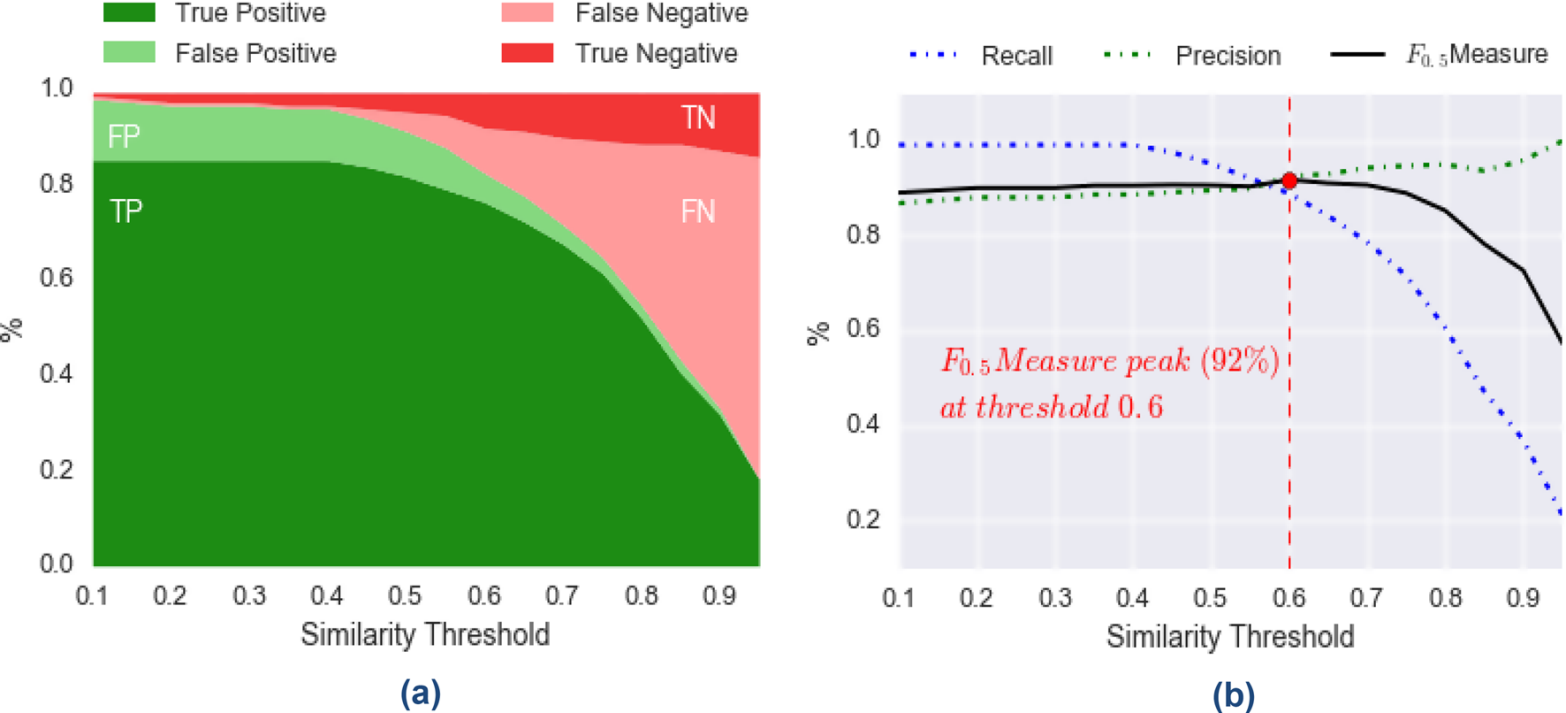
Seizure-agnostic algorithm performances. The seizure-agnostic algorithm performances and similarity thresholds show 92% precision values. In (**a**) we show how the distribution of True-Positives (TP), False-Positives (FP), True-Negatives (TN), False-Negatives (FN) change according to different values of the similarity threshold (within the range [0.1, 1]). Similarly, in (**b**) we show how the derived performance metrics (precision, recall, and F_0.5_-measure) change with respect to the similarity threshold. We note a peak of F_0.5_-measure (92%) at threshold equal to 0.6, for which we get precision equal to 92%, and recall equal to 89%.

Let us consider the seizure-agnostic algorithm. Figure 3 reports on the output of our experiments. In particular, in (a) we show how the distributions of False/True Positives and False/True Negatives change with respect to the threshold value considered, while in (b) we show how performance metrics change accordingly to such distributions. As one can expect, True-Positives decrease as the threshold value increases, as does precision. Setting a high threshold value leads the algorithm to focus only on strongly related samples, but we may be able to discard some correlations. As a matter of fact, as threshold value increases the number of matches found (the sum of True and False Positives) drastically decreases. This fact is also reflected by recall trend, which is stable at *100%* from *0.1* to *0.4*, but it then drastically decreases as well. A good balance of precision and recall is found at threshold equal to *0.6*, where F_0.5_-measure is maximum (*92%*), precision is *93%*, and recall is *89%*.

**Figure 4:**
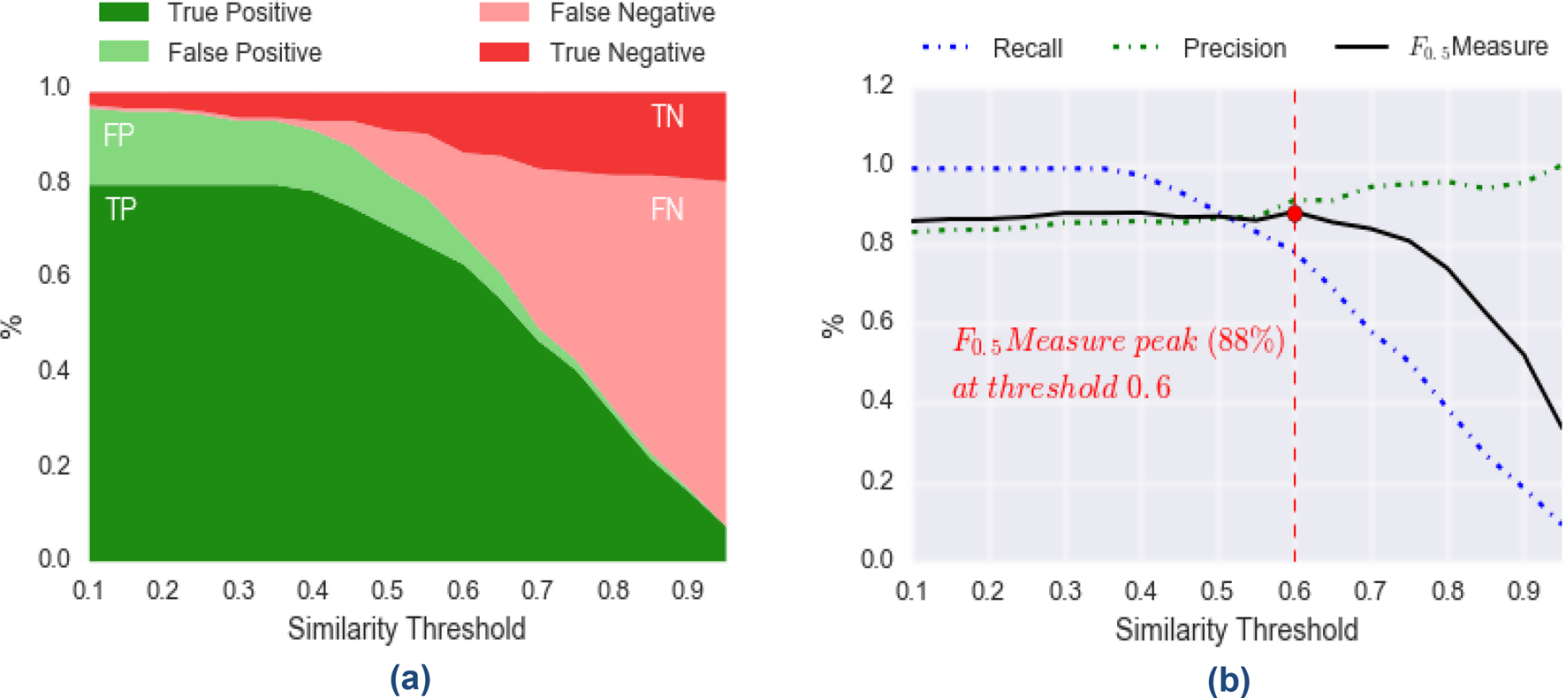
Seizure-aware algorithm performances. The seizure-aware algorithm performances and similarity thresholds show 91% precision values. In (**a**) we show how the distribution of True-Positives (TP), False-Positives (FP), True-Negatives (TN), False-Negatives (FN) change according to different values of the similarity threshold (within the range [0.1, 1]). Similarly, in (**b**) we show how the derived performance metrics (precision, recall, and F_0.5_-measure) change with respect to the similarity threshold. We notice a peak of F_0.5_-measure (88%) at a threshold equal to 0.6, for which we get precision equal to 91%, and recall equal to 78%.

Analogously, if we consider the performances of the seizure-aware algorithm (Fig. 4), we see that we find the maximum value of F_0.5_-measure (*88%*) at a threshold of *0.6*, where precision is *91%*, and recall is *78%*. Note that we have a small reduction of recall with respect to the seizure-agnostic algorithm, although it is still reasonably high.

These performances lead us to consider our algorithms as sufficiently reliable and can thus be used to help law enforcement providing useful insights on cocaine production history. Nevertheless, further experiments may be needed in order to fully refine our algorithm. Indeed, we may test them against heterogeneous large-scale datasets, which might show us possibly different ranges of performances, while suggesting further refinements to the algorithms. Moreover, if we could have access to investigation data, we may be capable of building a more reliable gold standard to better evaluate performances and improve the engineering of similarity features.

A large dataset of chemical analysis of drugs, shared in real time across Europe, is not currently available. Frequently, data is used regarding local or small-scale drug supply monitoring networks.

For this instance, another important accomplishment of this research was the creation and development of the first computerized, web-based tool for data collection, mining, and inference of cocaine seizures and trafficking market trends. This also allowed the implementation of coordinated efforts for communication and information exchange across European law enforcement operators.

The PRIDE system is now an innovative system which has the ability to collect, relate, infer, and visualize data on a map and retain specific information about cocaine seizures in a permanent database. The system includes an external link to upload non-confidential data such as chemical analytical results (concerning main active principles and impurities having toxic effects on health) downloadable from authorized healthcare providers to promote targeted drug preventive actions.

Furthermore, detailed information about the structure, manufacturing processes and variations of drug trafficking can be constantly updated and shared in real time. By doing this, the system can be used to understand links between samples, identify places of packaging and trafficking routes, and to detect sources of supply and common origins between seizures.

During the research, variations in chemical properties between samples coming from seizures with different origins were noticed (i.e. metal levels, especially lanthanide groups). Variations in profiles of illicit drugs synthetized in the same laboratory, however, were assumed to be relatively small.

Consequently, parameters used for comparison and linkage- dependent on laboratory and production processes- could potentially be measured regarding similarity between specimens according to a statistical method. Chemical, genetic and physical profiling of illicit drugs can be considered a systematic process in which specimens are compared with existing data that is already organized and stored. Sample references can be interpreted in crime situations and provide factual and objective information to intelligence bodies.

Data storage for instance, will provide support to the limitations of seizure’s representativeness. Data is pre-organized in the memory of the system- the core part of the database- with groups of similar chemical profiles that are created according to chemical, physical, or genetic profiles. This will allow authorities to recognize links between the new specimen when it is registered and existing data. This data will be constantly updated so it is aligned with the illicit drug market’s rapid evolution (as comparisons between recent profiles with those collected long time ago would probably be irrelevant).

In this way, the PRIDE system located on a web platform is a great contribution to the coordination of drug supply reduction at international, national and sub-national levels. The system is user-friendly and offers access to an unlimited number of authorized users at any given time (law enforcement officers, customs agencies, etc.). Moreover, the system processes data concerning chemical analyses of drugs and information about seizures, thus inferring useful results for law enforcement activities.

A time series of cocaine seizures, found in any location of the Italian territory, has been included in this study. Inferential elaborations concerning entity, origin, and direction of the drug market starting from similarities/differences among chemical profiles and characteristics of the seizures have been made.

Maximum information has been obtained using the following analytical method:

1. Selection of compound groups able to characterize cocaine seizures as volatile organic compounds (residual solvents), non-volatile organic compounds (origin impurities, by-products);
2. Quantitative detection of cocaine by gas chromatographic detection and origin impurities (minor compounds of coca leaves such as cis and trans cinnamoylcocaine, tropococaine, hydroxycocaine, trimetoxycocaine, truxillines) detection by a general unknown analysis performed by gas chromatography–mass spectrometry (GC/MS) in scan mode. This approach also planned a computation of the ratio of each substance detected against cocaine and against cinnamoylcocaine.
3. Detection of volatile organic compounds for determination of residual solvents has been performed using GC/MS using solid-phase microextraction (SPME) technique.

## Methods

Research activities to create the PRIDE platform for relating cocaine seizures included laboratory analyses investigations on cocaine samples and the design and implementation of the PRIDE platform. This paragraph on methods will be divided into two sections concerning each of the above-mentioned steps of the research.

### Laboratory analyses on cocaine seizures

Chemicals and solvents used for laboratory analyses on cocaine samples were reagent grade or better.

For each solvent examined (toluene, ethyl acetate, methyl cyclohexane, pentane, propyl acetate, acetone, dichloromethane, methanol, heptane and xylene), a stock solution with appropriate dilution in DMSO (Sigma-Aldrich, Steinheim, Germany) was prepared. Acetone D6 (VWR, Radnor, Pennsylvania, USA) was used as an internal standard for the quantitative detection of acetone, pentane, dichloromethane and ethyl acetate; toluene D8 (Acros, Thermo Fisher Scientific, San Jose, CA, USA) was employed as an internal standard for the quantitative determination of propyl acetate, heptane, methyl cyclohexane, toluene and xylene. A stock internal standard solution (ISS) was prepared by diluting both deuterated solvents in DMSO at the following concentrations: acetone D6, 6.581 µg/mL and toluene D8, 7.117 µg/mL.

Standards of cocaine, methylecgonine (EME), ethylecgonine (EEE), tropacocaine, norcocaine, cocaethylene, and n-formyl norcocaine were purchased from Cerilliant (Sigma Aldrich, Round Rock, Texas, USA). A stock standard solution was prepared at a concentration of 0.1 mg/mL in methanol containing all the compounds. Ethaverine 0.2 mg/mL in methanol was used as an internal standard for quantitative determination.

The extraction of occluded solvents was performed by a solid-phase microextraction (SPME) device equipped with a 100 µm PDMS (Polydimethylsiloxane) fused silica fibre (Supelco, Bellafonte, PA, USA) in the head space modality (HS-SPME).

Gas chromatographic–mass spectrometric (GC–MS) analyses were performed on a Trace 1300 GC (Thermo Fisher Scientific, San Jose, CA, USA) coupled with ISQ quadrupole mass spectrometer (Thermo Fisher Scientific, San Jose, CA, USA). The separation of organic volatile compounds was carried out by means of a DB-1 capillary column (60 m × 0.25 mm × 1 µm) from Agilent (Santa Clara, CA, USA); alkaloids and by-products were analyzed by means of a TG-5 MS capillary column (15 m × 0.25 mm × 0.25 µm) from Thermo Fisher (San Jose, CA, USA). The purity of cocaine was performed by GC analysis with flame ionization detector (FID) (Thermo Fisher Scientific, San Jose, CA, USA) using a TR-5 capillary column (7 m × 0.32 mm ID × 0.25 µm).

### Drug samples collection and preparation

Specific permissions have been obtained from governmental authorities to collect cocaine samples coming from big seizures (> 5 kg). Sampling has been made according to the European guidelines and internal rules (TDT Project Sampling Protocol). Forty exhibits that were seized in the main harbours of Italy between 2013 to 2017 were analyzed in order to characterize the differences among seizures through the identification and quantification of cocaine, origin and by-product impurities, and volatile organic compounds. The samples were mainly constituted by rectangular loaves (about 1 kg each) wrapped in cellophane and rubbery material with different logos (i.e., stars, moons, spheres).

All the specimens appeared as white material, fine or granulated powder, or sometimes as sticky matter. The sampling was performed according to the international guidelines of the “Scientific Working Group for the Analysis of Seized Drugs” (SWGDRUG Recommendations, 2016-June-9, www.swgdrug.org, accessed September 2017) that were specifically designed for such experiments. Various samplings from each exhibit were taken, depending on the amount of the seized powders, the wrapping of the package, the presence of different logos, the features of the material. A total of one hundred forty-eight samples were collected from random packages.

The analyses were performed during 2017 and 2018 in the laboratories of Forensic Toxicology at the Catholic University of Sacred Heart of Rome.

Sample treatment is a fundamental step in the analytical methodologies. Different extraction procedures were applied to cocaine samples in order to detect cocaine purity, origin and by-products impurities content and occluded solvents.

Approximately 30 mg of illicit cocaine powders were accurately weighted and diluted with 10 mL of IS solution (ethaverine 2.0 mg/mL in methanol). The solution was directly injected in GC-FID for the determination of cocaine purity. Successively, the solutions were appropriately diluted with LC-grade methanol to achieve a suitable concentration for GC/MS detection.

In order to determine the occluded solvents, approximately 200 mg of cocaine powder were accurately weighted into a 20 mL headspace vial and diluted with 4.3 mL of DMSO and 1 mL of saturated NaCl aqueous solution; 20 µL stock solution of both internal standards (acetone D6 and toluene D8) were added, and the vials were then sealed. HS-SPME was carried out using static headspace mode as follows: after 5 min of equilibration at 70 °C, residual solvents were sampled for 15 min at the same temperature. The fibre was then exposed in the GC injector at 150 °C for 2 min, to permit the desorption of the analytes.

### GC–MS analysis

For the complete characterization of the chromatographic profile of volatile compounds, GC–MS analysis in total ion current (TIC) acquisition mode was performed on the seized cocaine samples. We were able to identify twenty-seven solvents by means of comparison with a specific MS database, then confirmed the identification with appropriate standards. The most commonly found solvents in illicit powders were chosen for the validation: acetone, pentane, dichloromethane, ethyl acetate, propyl acetate, heptane, methyl cyclohexane, toluene, m-xylene, o-xylene and p-xylene; hexane and benzene were excluded due to their high toxicity.

The analyses were then carried out by GC–MS in single ion monitoring (SIM) mode, selecting the target ion for each solvent using the IS method (IS: toluene D8 and acetone D6).

The setting parameters for the mass spectrometer were ionization through electron impact at 70 eV and an ion source at 200 °C. The GC temperature program steps were applied as follows: from 50 °C (1 min isotherm) up to 80 °C at a rate of 30 °C/min, then to 150 °C at a rate of 10 °C/min, and finally up to 240 °C at a rate of 20 °C/min (5 min of final isotherm). Other GC parameters were a split injection ratio of 1:12.5, helium as carrier gas at a flow rate of 1.2 mL/min, injector set at 150 °C, purge flow 5.0 mL/min.

The determination of origin and by-products impurities was performed by GC–MS in TIC mode using the internal standard method (IS ethaverine). The following compounds were selected: methyl ecgonine, ethyl ecgonine, tropacocaine, norcocaine, cocaethylene, and n-formyl norcocaine.

The chromatographic separation was carried out by means of the following GC temperature program: from 80 °C (2 min isotherm) up to 270 °C at a rate of 15 °C/min, then to 300 °C at a rate of 50 °C/min (5 min final isotherm). Other GC parameters were split injection ratio of 1:12.5, helium as carrier gas at a flow rate of 1.2 mL/min, injector set at 150 °C, purge flow 5.0 mL/min. The quantitative determination of the substances was performed using the extracted ion current method (XIC) linked with their specific target ions.

Both GC–MS methods were validated according to the recommendations of SWGDRUG. Selectivity, limit of detection (LOD), lower limit of quantification (LLOQ), linearity, accuracy, and repeatability (intra-day and inter-day precision) were evaluated to ensure the acceptability of the analytical procedure.

### Validation parameters

Regarding the occluded solvents, the selectivity was evaluated by ten different powder samples of mannitol, usually employed to dilute illicit preparations. It was investigated with and without internal standards to check for peaks that might interfere with the detection of the selected solvents. LOD and LLOQ were estimated from the signal-to-noise ratio (3:1 for LOD while 10:1 for LLOQ).

The linearity of the method was studied with the internal standard method using ten different concentrations, including LOD and LLOQ. Working standard solutions were prepared at different levels of concentration for each solvent examined and added with a fixed internal standard (IS) concentration (7.117 µg/mL for toluene D8 and 6.581 µg/mL for acetone D6).

The calibration curves were found to be linear (correlation coefficient equal to or greater than 0.99) over a wide range of concentrations; correlation coefficients (R^2^) and range of linearity are reported in Table 4. The LOD values demonstrated high sensitivity in all of the substances (except ethyl acetate 338.50 ng/mL) varying from 1.30 ng/mL for heptane to 29.4 ng/mL for methyl cyclohexane. The LLOQ values were estimated to range from 2.60 ng/mL for heptane to 67.2 ng/mL for propyl acetate; acetone and ethyl acetate showed higher LLOQ (Table 4). These values guarantee the detection of the residual solvents in cocaine powders. Moreover, the method showed high selectivity and no interferences were detected.

**Table 4:** Validation parameters for volatile organic compounds.

Residual solvent	LOD (ng/mL)	LLOQ (ng/mL)	Linearity (µg/mL)	R^2^	Mean concentration ± SD (n = 5)	Intra-assay precision (n = 5 runs)		Mean concentration ± SD (n = 5)	Inter-assay precision (n = 5 runs)	
						Mean relative error (%)	RSD		Mean relative error (%)	RSD
Acetone	14.90	149.20	LLOQ-14.92	0.9957	149.0 ± 0.31	9.18	9.53	148.9 ± 0.26	10.93	8.18
Penthane	23.60	47.20	LLOQ-11.81	0.9939	47.0 ± 1.16	21.73	10.76	47.0 ± 0.61	16.60	31.59
Dichloromethane	25.10	50.20	LLOQ-25.09	0.9999	50.2 ± 0.90	23.32	11.09	50.1 ± 1.29	24.97	17.23
Ethyl acetate	338.50	846.20	LLOQ-84.62	0.9992	846.1 ± 0.98	32.95	12.36	846.0 ± 1.62	32.77	16.93
Propyl acetate	16.80	67.20	LLOQ-16.79	0.9994	67.1 ± 1.20	13.60	13.65	67.1 ± 1.11	13.58	12.86
Heptane	1.30	2.60	LLOQ-1.30	0.9982	2.6 ± 0.03	13.52	4.54	2.6 ± 0.1	13.85	6.53
Methyl cyclohexane	29.40	59.00	LLOQ-1.47	0.9944	59.1 ± 0.66	13.60	13.13	29.4 ± 0.74	5.32	13.27
Toluene	3.27	32.70	LLOQ-16.36	0.9999	32.7 ± 0.02	5.15	7.91	32.6 ± 0.03	5.32	13.02
o-Xylene	6.50	32.60	LLOQ-1.63	0.9999	3.3 ± 0.17	16.67	19.63	3.3 ± 0.42	18.44	15.16
m-Xylene	6.50	32.60	LLOQ-1.63	0.9998	3.3 ± 0.17	20.27	16.82	3.3 ± 0.14	23.30	14.10
p-Xylene	6.50	32.60	LLOQ-1.63	0.9994	3.3 ± 1.04	18.82	27.85	3.3 ± 0.07	22.05	18.81

Results of the calibration curve for the detection of residual solvents in cocaine powders. The linearity of the method was studied with the internal standard method using ten different concentrations, including LOD and LLOQ.

Repeatability (intra-day assay) and intermediate precision (inter-day assay) were also evaluated. The repeatability was studied using 25 independent standard solutions containing all solvents at their LLOQ concentration level. To evaluate the intra-day precision, five independent samples in a day were examined; for intermediate precision, five samples for five days were analyzed. The results obtained were expressed as a coefficient of variation (CV%).

According to FDA criteria [US Department of Health and Human Service, Food and Drug Administration, Centre for Biologics Evaluation and Research (2001) Guidance for Industry: Bioanalytical Method Validation https://www.fda.gov/downloads/Drugs/GuidanceComplianceRegulatoryInformation/Guidances/UCM070107.pdf (accessed September 2017)], precision and accuracy should not exceed 15%, except for the LLOQ, where it should be no more than 20%. The accuracy and precision of the cocaine samples herein studied were determined by intra-day and inter-day replicated analyses (Table 4).

We obtained acceptable precision and accuracy (calculated at LLOQ) always lower than 25%, except for ethyl acetate. Although not within the FDA’s criteria, we retain that values obtained should be accepted for the chemical physical properties of the compounds herein studied due to their high volatility.

Regarding the validation of the selected impurities, selectivity, LOD, and LLOQ were estimated using the method previously described for occluded solvents. The linearity was also studied using the internal standard method. Working standard solutions were prepared at five levels of concentration for each of the six impurities investigated (methyl ecgonine, ethyl ecgonine, tropacocaine, norcocaine, cocaethylene, and n-formyl norcocaine); they were added with a fixed internal standard (IS) concentration (ethaverine 0.2 mg/mL in methanol) and calibration curves were prepared in triplicate. The calibration curves, considered linear when the correlation coefficient (R^2^) was equal to or greater than 0.99, covered a wide range of concentrations; R^2^ and range of linearity are shown in Table 5. Sensitivity, LOD, and LLOQ values found for each compound are also included in the same table. The LLOQ values were estimated to be as low as 3.84 ng/mL for all the impurities except for the tropacocaine (1.96 ng/mL). These LLOQ values demonstrated high sensitivity of the method.

**Table 5:** Validation parameters for alkaloids.

Alkaloid	LOD (ng/mL)	LLOQ (ng/mL)	Linearity (µg/mL)	R^2^	Mean concentration ± SD (n = 5)	Intraassay precision (n = 5 runs)		Mean concentration ± SD (n = 5)	Inter-assay precision (n = 5 runs)	
						Mean relative error (%)	RSD		Mean relative error (%)	RSD
Ecgonine methyl ester	1.96	3.84	LLOQ-16.67	0.9936	5.50 ± 0.11	4.10	9.07	5.02 ± 0.38	14.36	1.51
Ecgonine ethyl ester	1.96	3.84	LLOQ-16.67	0.9934	5.50 ± 0.19	8.41	6.93	5.02 ± 0.24	8.71	7.26
Tropacocaine	0.93	1.96	LLOQ-15.67	0.9931	5.26 ± 0.27	6.88	4.63	4.84 ± 0.30	8.16	2.57
Norcocaine	0.99	3.84	LLOQ-16.67	0.9970	5.59 ± 0.28	9.39	7.23	5.38 ± 0.37	11.09	3.11
Cocaethylene	0.99	3.84	LLOQ-16.67	0.9926	5.45 ± 0.14	4.52	6.39	5.07 ± 0.23	7.95	4.36
N-formyl norcocaine	0.99	3.84	LLOQ-16.67	0.9918	5.60 ± 0.46	12.24	7.55	5.29 ± 0.56	15.96	1.30

Results of the calibration curve for the detection of impurities, by-products. The selectivity, LOD, and LLOQ were estimated using the same method used for occluded solvents. The linearity was also studied using the internal standard method.

Repeatability (intra-day assay) and intermediate precision (inter-day assay) were evaluated with the same method applied to occluded solvents as previously described. The method showed high selectivity and no interferences were detected. The accuracy and precision determined by intra-day and inter-day replicated analyses are reported in Table 5. All results obtained agree with FDA criteria.

### Creation of the algorithm

In this section, we present the seizure-agnostic algorithm. We remark that the seizure-aware algorithm is just a simple variation of the seizure-agnostic algorithm, where we discard the samples in the same seizure of a given sample *i*.

**Figure 5:**
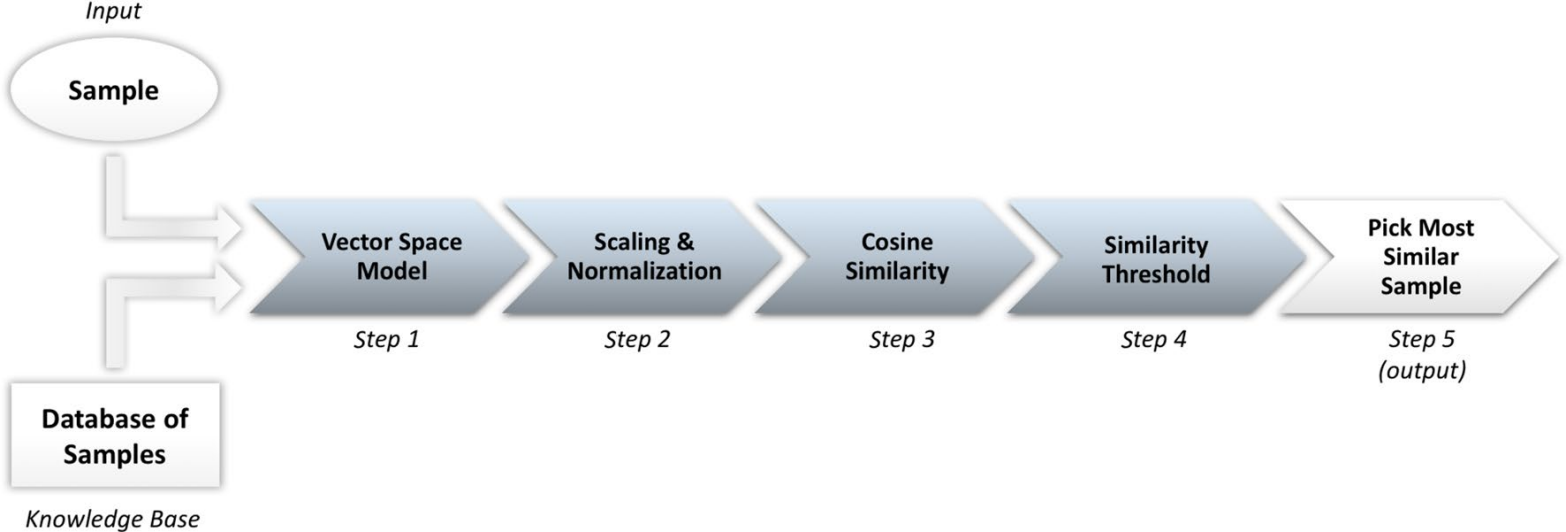
The algorithm. A diagram of the algorithm explains the input, output and the 5 steps necessary. Given a sample *i*, it is added to the database, and we then compute our Vector Space Model (Step 1). In this space, each dimension represents a similarity feature identified by a compound in Table 1, thus we use 35 dimensions. The value for dimension *x,* in a vector *A* representing sample *i,* is the concentration of compound *x* for sample *i* (Figure S1a). After this, we normalize and scale all vectors (Step 2), in order to obtain a new VSM where vectors have magnitude 1, are centered on the mean, and have component-wise unit variance (Figure S1b). We experimentally verified that such transformations have important effects on the prediction reliability of our algorithm. (To see Supplementary Figure S1, please access Supplementary Information). Once we have such a VSM, we can compute similarity values between the vector *A,* corresponding to the given sample *i,* and all other vectors (Step 3). Given two vectors *A* and *B*, representing, respectively, samples *i* and *j*, we compute their similarity as the cosine of the angle between *A* and *B* (Figure S2), i.e., their cosine similarity. In our VSM, where all of the vectors have magnitude *1*, the cosine similarity can be easily computed as the dot product of *A* and *B*. After computing all of the similarity values between the given sample *i* and all of the other samples, we discard those samples showing a similarity less than a pre-defined threshold, *th* (Step 4). We remark that *th* has been set to *0.6*, according to the experiments discussed in the text. If there are samples with a similarity value higher than equal to *th*, we pick the one with the highest similarity value among them (Step 4), otherwise we conclude that we found no similar sample to *i*. If a similar sample is found, we output on the data and metadata of the selected sample and related seizure. (To see Supplementary Figure S2 Cosine similarity, please access Supplementary Information).

The main steps of our algorithm are depicted in Fig. 5.

Given a sample *i*, it is added to the database, and we then compute our Vector Space Model (VSM) (Fig. 5, Step 1). In this space, each dimension represents a similarity feature identified by a compound in Table 1, thus we use 35 dimensions. The value for dimension *x* in vector *A* is the concentration of compound *x* for sample *i* (Figure S1a). After this, we normalize and scale all vectors (Fig. 5, Step 2), in order to obtain a new VSM where vectors have magnitude 1, are centered on the mean, and have component-wise unit variance (Figure S1b). We experimentally verified that such transformations have important effects on the prediction reliability of our algorithm. (To see Supplementary Fig. S1, please access Supplementary Information). Once we have such a VSM, we can compute similarity values between vector *A,* corresponding to the given sample *i,* and all other vectors (Fig. 5, Step 3). Given two vectors *A* and *B*, representing, respectively, samples *i* and *j*, we compute their similarity as the cosine of the angle between *A* and *B* (Figure S2), i.e., their cosine similarity. In our VSM, all of the vectors have magnitude 1, the cosine similarity can be easily computed as the dot product of *A* and *B*. After computing all of the similarity values between the given sample *i* and all of the other samples, we discard those samples which show a similarity less than a pre-defined threshold, *th* (Fig. 5, Step 4). We remark that *th* has been set to *0.6*, according to the experiments previously discussed. If there are samples with a similarity value higher than or equal to *th*, we pick the one with the highest similarity value among them (Fig. 5, Step 4). Otherwise, we conclude that we found no similar sample to *i*. If a similar sample is found, we output on the data and metadata of the selected sample and related seizure. (To see Supplementary Fig. S2. Cosine similarity, please access Supplementary Information).

As you may notice, when creating the design of the algorithm, we selected well-known machine learning techniques and combined them to get an algorithm, which should be simple to maintain, analyze, and extend. Indeed, we remark that we designed this algorithm in order to show the possibility of successfully applying machine learning techniques to this field, while finding the best possible algorithm for this specific application is beyond the scope of this paper. In addition, looking for a better algorithm requires a vast dataset of labeled data and further experiments. The lack of such an amount of data thus limited the extent of our research. Therefore, we encourage further research to be done in order to refine the algorithms, test them on more and more data, and try to extend the entire methodology to other fields of interest.

## Conclusions

Illegal drug trafficking imposes significant threats to public health in terms of premature mortality, lost productivity, as well as other intangible costs in the form of poor quality of life for drug consumers. Increased intelligence efforts are key in the process of reducing illegal drug trafficking. In this research, a new system of classification of drugs deploying machine learning was presented, making a prototype available for drug investigations and control across Europe.

To create the prototype, cocaine has been chosen as one of the most investigated and widespread illegal drugs. That being said, the PRIDE system can also be applied to highly variable drugs which are the emerging challenges for its future applications.

This is the first report on using machine learning for automatic classification of cocaine drug profiles. Compared with manual classification, the results of this study demonstrated that PRIDE, a machine learning system, offers the possibility of faster and broader scale usage compared with current manual procedures, as well as the possibility of a gold standard and systematic procedure to be developed in the area. The model achieved values of 88% for F_0.5_-measure, 91% for precision, and 78% for recall. The study successfully confirmed the possibility of a machine learning application for automatic classification of cocaine profiles. Other extended uses will be possible as well as other algorithmic techniques as they become integrated, leading to even better results.

## Supplementary information

Supplementary information.

## Data Availability

All data generated or analyzed during this study are included in this published article (and its Supplementary Information files).
