# Supplementary material for: A data-driven methodology to discover similarities between cocaine samples
Source: Sci Rep. 2020 Sep 29;10:15976. doi: 10.1038/s41598-020-72652-w (PMC7525495; doi:10.1038/s41598-020-72652-w)
Supplement: Supplementary file 1 — Supplementary information. [file 41598_2020_72652_MOESM1_ESM.docx]

SUPPLEMENTARY ONLINE MATERIAL

**A data-driven approach to discover similarities between cocaine samples.**

**Author list and affiliations**

Fidelia Cascini^1^*, Nadia De Giovanni^2^, Ilaria Inserra^1^, Federico Santaroni^3^, Luigi Laura^3^.

^1^ Institute of Public Health, Section of Forensic Medicine, Catholic University of the Sacred Hearth, 00168 Rome, Italy

^2^, Fondazione Policlinico Universitario A. Gemelli IRCCS, Largo Agostino Gemelli 8 – 00168, Roma, Italia

^3^ Department of Computer, Control, and Management Engineering Antonio Ruberti (DIAG), Sapienza University of Rome, 00186 Rome, Italy

**Correspondence to:** Professor Fidelia Cascini

Institute of Public Health,

Section of Forensic Medicine,

Catholic University of the Sacred Hearth

00168 Rome, Italy

Email: [fidelia.cascini1@unicatt.it](mailto:fidelia.cascini1@unicatt.it)

**Supplementary information**

**FIGURES**


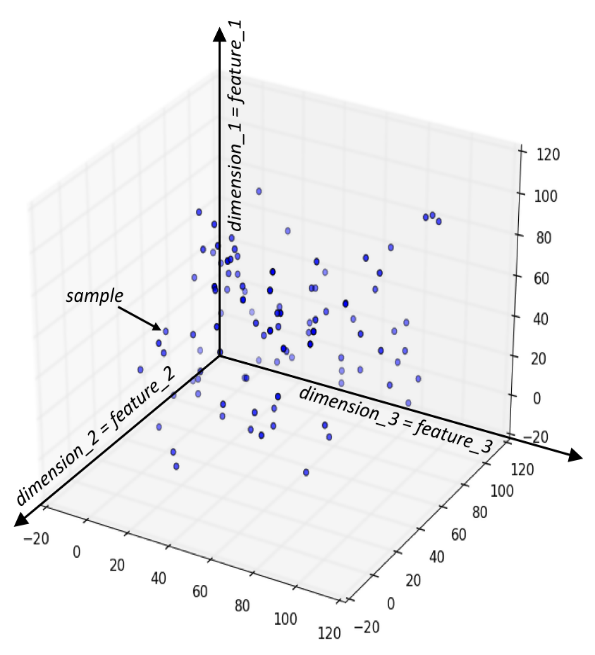

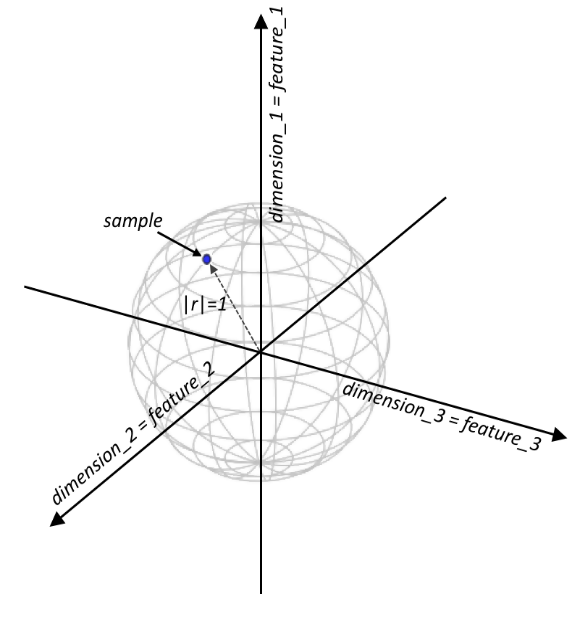


**(a)**

**(b)**

**Supplementary figure S1. Vector space model**

Figure S1. For the sake of representation, we depict the VSM as three-dimensional, but in our actual VSM we use 35 dimensions, i.e., the number of compounds listed in Table 1. In (a), we depict a VSM where samples are encoded as vectors in a multi-dimensional space, where each dimension corresponds to a similarity feature (compound concentration). We then scale and normalize our VSM (b), obtaining vectors pointing on the surface of a hyper-sphere of radius 1.


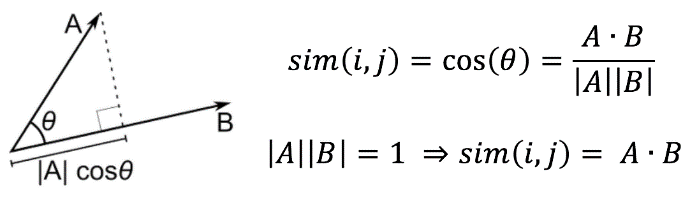


**Supplementary figure S2. Cosine similarity**

Figure S2. Let A, B be two vectors, and θ the angle between them. In order to have the same direction, the two vectors must have the same distribution of values in their underlying dimensions. Note that in our VSM all vectors have magnitude 1, thus the cosine similarity can be efficiently computed as the dot product between A and B. In addition, in these settings, a similarity of 1 means the two vectors are completely coincident.

**DISCUSSION**

**Domain expert approach:**

- We say that a compound is present in a sample if its concentration is over its measurement tolerance, which depends on the instruments used for the analysis phase.
- Two samples, to be considered similar, should show the same set of compounds to be present in both of them.
- If the two sets of compounds found in the given pair of samples differ for more than two compounds, we conclude that the two pair of samples may not be similar.
- If the two sets of compounds found in the given pair of samples differ for less than equal two compounds, we consider the intersection of the two sets, i.e., the set of compounds present in both samples:
  - For each compound, if the concentration measured differs for at most 25%-30%, they are considered similarly distributed.
  - If all the compounds are similarly distributed, we may conclude that the samples are similar.
  - Otherwise, if there are compounds which are not similarly distributed, we consider the two samples as potentially similar, but we might have not enough evidences of this fact.
